# Eye findings and immunological markers in probands and their euthyroid relatives from a single family with multiple cases of thyroid autoimmunity

**DOI:** 10.1186/1756-6614-5-4

**Published:** 2012-06-28

**Authors:** Melissa Ardley, Thomas McCorquodale, Hooshang Lahooti, Bernard Champion, Jack R Wall

**Affiliations:** 1 The Department of Medicine, The University of Sydney, Nepean Hospital, PO Box 63, Penrith, NSW 2751, Australia; 2 University of Sydney, Western Clinical School, Nepean Hospital, PO Box 63, Penrith, NSW 2751, Australia

**Keywords:** Thyroid autoimmunity, Ophthalmopathy, Graves’ disease, Genetics, Euthyroid relatives, Calsequestrin

## Abstract

**Background:**

Ophthalmopathy is a common manifestation of Graves’ disease (GD) occurring in up to 50% of patients. Mild eye signs are also common in patients with Hashimoto’s thyroiditis. Whilst a genetic predisposition to GD has been demonstrated this is not the case for the ophthalmopathy which often runs a separate course.

**Objective:**

We determined the prevalences of eye and eyelid signs and positive thyroid and orbital antibody tests in first and second degree relatives from a single family with multiple cases of Graves’ disease, ophthalmopathy and Hashimoto’s thyroiditis.

**Design:**

The study cohort comprised 16 subjects from the same family, 4 probands namely, 3 with GD and one with Hashimoto’s thyroiditis and hypothyroidism and 12 of their euthyroid first or second degree relatives. We measured antibodies against calsequestrin (CASQ1) and collagen XIII in an enzyme-linked-immunosorbent assays and TSH-Receptor (TSH-R) antibodies as i) TSH-R binding inhibiting immunoglobulin (TBII) and ii) thyroid stimulating immunoglobulin (TSI). Eye signs were classified and quantified using the clinical activity score (CAS), NOSPECS classes, Nunery types 1 and 2 and the margin-reflex-distance (MRD) as a measure of upper eyelid retraction (UER).

**Main outcomes:**

Whilst significant ophthalmopathy was uncommon in the relatives, mild eye signs, in particular UER, were demonstrated in about a third of them. The presence of eye signs was moderately, but not significantly, associated with the detection of CASQ1 and collagen XIII antibodies, but not TSH-R antibodies.

**Conclusion:**

Our study demonstrates a significant prevalence of positive orbital antibody tests and ophthalmopathy in probands with thyroid autoimmunity and their euthyroid relatives, favouring a role of genetic factors in the development of ophthalmopathy in patients with thyroid autoimmunity.

## Introduction

Graves’ disease (GD) is the most common form of hyperthyroidism, affecting up to 1% of the population [1]. There is a marked gender differences in prevalence with a 5–10 fold excess in females and a significant clustering of disease in families, with 40-50% of patients reporting at least one family member with a thyroid disorder [2]. GD is considered to be a heterogeneous autoimmune disorder affecting, with varying degrees of severity, the thyroid, eyes and skin [3]. McKenzie [4] has defined GD as “a multi system disorder of unknown aetiology, characterized by one or more of three clinical entities; 1) hyperthyroidism associated with diffuse hyperplasia of the thyroid gland 2) infiltrative ophthalmology and 3) infiltrative dermopathy (localized pretibial myxedema)”.

GD is a classical autoimmune disorder in which targeting of thyroid antigens, in particular the thyroid stimulating hormone (TSH) receptor (TSH-R), by antibodies and sensitized T lymphocytes leads to proliferation of thyrocytes and overproduction of thyroid hormone. There is a genetic susceptibility to autoimmunity which is triggered by unknown environmental factors [5,6]. Recent studies have identified several genetic susceptibility loci including human leukocyte antigen (HLA, 6p21.3) and cytotoxic T-lymphocyte antigen-4 (CTLA-4, 2q33) [7]. Several environmental factors have also been elucidated including cigarette smoking, psychosocial stress, iodine intake, intrauterine growth, bacterial and viral infections and drugs such as interferon [8].

Ophthalmopathy is a common manifestation of autoimmune thyroid disease occurring in about 50% of patients with GD where it is called Graves’ ophthalmopathy (GO) [9], and in about 25% of patients with Hashimoto’s thyroiditis [10]. Ophthalmopathy may develop as a result of cross-reaction against a thyroid and orbital tissue antigen(s) in orbital connective tissue and eye muscle. Candidate orbital connective tissue antigen include the TSH-R, which is also expressed in the orbital fat and pre adipocytes [11-14] and possibly eye muscle as well [15], and the fibroblast cell membrane antigen collagen type XIII [16,17]. Of the several eye and eyelid muscle antigens identified [3] the skeletal muscle calcium binding protein calsequestrin (CASQ1) is the best candidate [17-19]. In order to further study the role of orbital antibodies in the pathogenesis of ophthalmopathy, and to address a possible genetic role, we have determined the prevalences of eye and eyelid signs and positive thyroid and orbital antibody tests in first and second degree relatives from a single family with multiple cases of thyroid autoimmunity.

## Materials and methods

### Clinical Subjects

**Figure 1:**
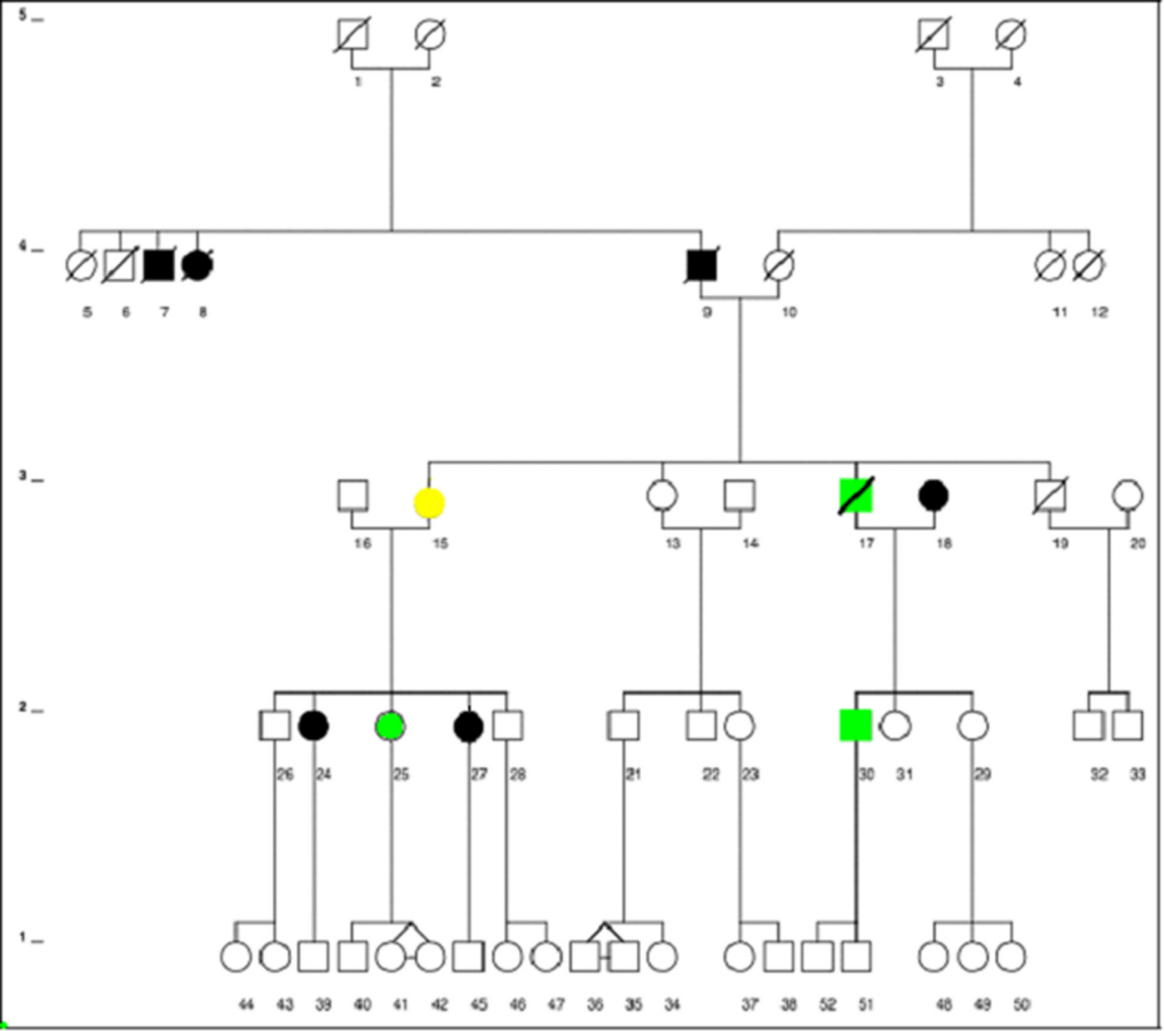
Family Tree showing probands with Graves’ disease or Hashimoto’s thyroiditis and their euthyroid relatives, 6 of whom had “thyroid disease of unknown aetiology”.

The study involved 16 subjects, 10 females and 6 males aged 13–76 (mean age 40 years) from the same family in Melbourne, Victoria (Figure 1). Within this branch of the family there are three cases of confirmed GD, both male, one case of Hashimoto’s thyroiditis and hypothyroidism and 12 first or second degree relatives of whom 6, 4 females and 2 males had a history of thyroid disease of unknown aetiology. Diagnosis of thyroid disease was based on standard clinical criteria and confirmed by thyroid function testing and thyroid antibody measurements. The grade, severity and activity of any ophthalmopathy were classified as; 1) Nunery types 1 (without restrictive myopathy) or 2 (with restrictive myopathy) [20] (2) as the clinical activity score (CAS) (0–10) of Mourits *et al.*[21] which is a measure of disease activity 3) Werner’s NOSPECS class [22] and 4) the upper eyelid margin-reflex distance (MRD) which is the distance between the centre of the pupillary light reflex and the upper eyelid margin with the eye in primary gaze, as a measure of upper eyelid retraction (UER). An MRD of > 5 mm, which correlates with an UER score of > + using our assessment protocol, is taken as significant UER. Local Ethical Committee approval was received for the study and informed consent of participating subjects was obtained.

### Serum antibody measurement

The presence and level of serum orbital antibodies in probands and their relatives were determined using an enzyme-linked immunosorbent assay (ELISA). This procedure has been described in previous publications by this laboratory [10,17-19] and is standard. The antigens used were i) highly purified rabbit skeletal muscle CASQ1 which shares 97% homology with human calsequestrin and ii) recombinant human collagen XIII. Results were expressed as optical density (OD) at 405 nM. A positive test was taken as an OD > the upper limit of the reference range, which was 194 for CASQ1 and 193 for collagen XIII. Serum TSH-R antibodies were measured as i) TSH-R binding inhibiting immunoglobulin (TBII) where a positive test was taken as a level of > 1.5 IU/L and ii) thyroid stimulating immunoglobulin (TSI) using fresh human thyroid cells as source of cyclic AMP (cAMP) following stimulation with test serum and a cAMP ELISA kit where a positive test was, taken as a %B/BO > 19%, following the manufacturers instructions and reference ranges.

### Statistical analysis

Differences in prevalences of orbital and TSH-R antibodies were determined using the Mann–Whitney test for a non parametric data. A P value of <0.5 was taken as significant in all assessments.

## Results

The study cohort comprised 16 subjects from a single family, 4 probands of whom 3 had GD and one had Hashimoto’s thyroiditis and hypothyroidism and 12 first or second degree relatives. We measured antibodies against CASQ1 and collagen XIII in an ELISA, and TSH-R antibodies in two assays, and eye signs were classified and quantified as described in methods.

### Prevalence of thyroid dysfunction

At the time of study, all patients and relatives were clinically well and euthyroid. Sera were available from 16 subjects namely, the 4 probands [WH-18, WH-25 and WH-30 who had GD] and WH-15 who had hypothyroidism due to Hashimoto’s thyroiditis], and 11 first or second degree relatives. Of these, 3 subjects had abnormal TSH levels namely, two probands; WH-15 with a high TSH, and WH-18 with a low TSH both of whom were receiving thyroxine replacement, and one of the untreated relatives, WH-28, who had a low TSH (Table 1). The other probands and relatives were biochemically and clinically euthyroid at the time of testing.

**Table 1:** Demographic data, biochemical and immunological findings and treatment of probands with thyroid autoimmunity and their first or second degree relatives

**Subjects**		**Age (range yr)**	**Sex (F/M)**	**Mean serum TSH**^**1**^**(mIU/L)**	**Positive TPO**^**2**^**antibodies**	**TSH-R Ab**^**3**^		**Treatment**^**4**^		
						**TBII**	**TSI**	**L-T**_**4**_	**RAI ATD**	
Probands	GD^5^ with ophthalmopathy (n = 3)	52-75	2/1	1.84	2	2	0	3	3	1
	Other thyroid disorders (n = 1)	77	1/0	6.52	1	0	0	1	0	0
Relatives	No documented thyroid disorders (n = 11)	13-54	7/5	1.26	2	0	1	0	0	0

^1^ TSH = thyroid stimulating hormone (normal range 0.3-4.0 mIU/L).

^2^ TPO = thyroid peroxidase (cut-off titre 100).

^3^ A positive test for thyroid stimulating antibodies (TSI) was taken as a % B/BO >19%, a positive test for TSH-R binding inhibitory immunoglobulin (TBII) was taken as >1.5 IU/Litre.

^4^ Number of patients treated with thyroxine (L-T_4_), ^131^I or antithyroid drugs (ATD).

^5^ GD = Graves’ disease.

### Thyroid antibodies

Serum from two of the three probands with previously documented GD namely, WH-25 and WH-30, and from the hypothyroid subject WH-15, was positive for TPO antibodies. It is interesting to note that WH-15 has a daughter (not tested) with Hashimoto’s thyroiditis. Although none of the relatives were clinically or biochemically hyperthyroid, two, WH-29 and WH-31, the daughters and sisters of two of the Graves’ probands (see Figure 1), were positive for TPO antibodies (Table 1).

TSH-R antibodies, as TBII and TSI, were measured in most of the subjects. Positive TBII tests were demonstrated in two probands with GD, WH-30, and WH-25 and TSI tests were borderline positive in one apparently normal first degree relative, WH-26. The prevalences of positive TSH-R antibodies in probands vs relatives with and without ophthalmopathy were not significantly different (Mann–Whitney tests, P = NS).

### Ophthalmopathy

Next, we examined the 16 probands and relatives for eye and upper eyelid abnormalities. The overall prevalence of any eye signs was 37.5%. Three (20%) of the subjects were current smokers. In more detail, two probands (WH-18, WH-21) and two second-degree relatives (WH-49, WH-50) had mild ophthalmopathy (CAS = 1) and one proband (WH-30) had severe ophthalmopathy (CAS = 5) of many years duration which required corrective eye muscle surgery. Four of the probands (all 3 probands with GD and WH-15 with Hashimoto’s thyroiditis) also had mild UER and WH-30 had moderate ocular muscle dysfunction with restricted lateral gaze of the left eye. The duration of eye signs in these subjects was difficult to determine as they did not recognize any particular eye symptoms. All 4 probands with autoimmune thyroid disease, WH-15 with hypothyroidism and WH-18, WH-25 and WH-30 with GD, had eye signs. The other relatives were normal (Table 2).

**Table 2:** Prevalence of eye signs in probands and first or second degree relatives from a single family with multiple cases of thyroid autoimmunity

**Subject**	**CAS**^**1**^	**UER**^**2**^	**Nunery types 1, 2**^**3**^	**NOSPECS classes**^**4**^
WH-15	1	Yes	1	1
WH-18	2	0	1	2
WH-25	2	0	1	2
WH-26	0	0		0
WH-28	0	0		0
WH-29	0	0		0
WH-30	5	Yes	2	4
WH-31	0	0		0
WH-32	0	0		0
WH-41	0	0		0
WH-42	0	0		0
WH-48	0	0		0
WH-49	1	2	1	1
WH-50	1	0	1	2
WH-51	0	0		0
WH-52	0	0		0

^1^ CAS = Clinical activity score (CAS) (0–10) as described by Mourits *et al* (see ref no. 21).

^2^ UER = Upper eyelid retraction, taken as a MRD >5 mm which corresponds to an UER score of > +.

^3^ see ref no [20].

^4^ see ref. no [22].

### Orbital antibodies

We measured CASQ1 and collagen XIII antibodies in serum from all 16 subjects; namely, 4 probands and 12 euthyroid first or second degree relatives. Two of the three probands with GD, WH-18 and WH-30 who was also positive for TBII (but not for TSI), were positive for CASQ1 antibodies and one, WH-30, for collagen XIII antibodies (Table 3). The subject who tested positive for both antibodies was WH-30, the proband with severe ophthalmopathy (see Table 2). Of the four euthyroid relatives with positive eye muscle antibody tests only two (one with reactivity against CASQ1, WH-41, and one with reactivity against collagen XIII, WH-29) were first-degree relatives of probands with autoimmune thyroid disease. The other two euthyroid subjects with positive antibody results, WH-49 and WH-50, were the children of WH-29, i.e. second-degree relatives of probands with GD (see Figure 1). One of the second-degree relatives WH-49, had positive CASQ1 and collagen XIII antibody tests while another, WH-50, had a positive collagen XIII antibody test only. There was a modest overall correlation between any eye signs and positive antibody reactivity against CASQ1 and/or collagen XII, I although this did not reach statistical significance for ether antibody (Mann-Witney test P = NS, P = NS respectively) (Table 3).

**Table 3:** Prevalence of CASQ1 and collagen XIII antibodies in probands and first or second degree relatives from a single family with multiple cases of thyroid autoimmunity

**Subjects**	**Subjects**	**Prevalence (%) of positive antibody reactivity**	
		**CASQ1**^**1**^	**Collagen XIII**
Probands	GD^2^ with ophthalmopathy	2 (66%)	1 (33%)
	(*n* = 3)		
	Other disorders	0	0
	(*n* = 1)		
Relatives	No documented thyroid disorders	2 (16%)	3 (25%)
	(*n* = 12)		

^1^ CASQ1 = skeletal muscle calsequestrin.

^2^ GD = Graves’ disease.

Finally, all subjects with Graves’ disease and two first-degree relatives reported the presence of other autoimmune disorders (results not shown).

## Discussion

Graves’ ophthalmopathy is a common disorder which can present with either hyperthyroidism or hypothyroidism or in the apparent absence of thyroid autoimmunity, so called euthyroid Graves’ disease [9]. The ophthalmopathy associated with Graves’ hyperthyroidism and, less often, Hashimoto’s thyroiditis [10] is presumed to be an autoimmune disorder of the extraocular muscles and orbital connective tissue. There is substantial evidence that the eye muscles are targets of the autoimmune reactions of “thyroid-associated ophthalmopathy” because more than 90% of Graves’ patients with or without clinical signs of ophthalmopathy have extraocular muscle enlargement by orbital imaging techniques [23].

In the present study we determined the prevalence of CASQ1 and collagen XIII antibodies in serum from probands and first or second degree relatives of a single family with multiple cases of thyroid autoimmunity. We observed a fairly close relationship between CASQ1 and collagen XIII antibodies and ophthalmopathy, eye signs including stare and lid lag being demonstrated in all but one subject with positive eye muscle antibodies, although this was not significant, possibly because of the small numbers of subjects studied. Two thirds of those with positive eye muscle antibodies and eye signs had documented thyroid disease, 3 with GD and one with hypothyroidism, whilst two of the six subjects with eye signs were second-degree relatives of three probands with GD. It is interesting to note that WH-49 had a TSH result at the upper limit of normal and detectable antibodies against CASQ1 and collagen XIII, making her an interesting subject for long term follow-up at risk to develop thyroid dysfunction and/or ophthalmopathy in the future.

Several groups [24-26] have shown that relatives of patients with Graves’ disease have an increased prevalence of thyroid antibodies compared with controls, as well as an increased tendency to develop other autoimmune diseases. The notion that autoimmunity against the TSH-R is involved in the development of GD and GO and evidence for increased thyroid antibodies in euthyroid relatives of patients with GD makes a strong case for the familial inheritance of orbital antibodies as well. While significant ophthalmopathy is uncommon in relatives of subjects with GD, mild eye signs, in particular upper eyelid abnormalities, associated with eye muscle antibodies against CASQ1 and collagen XIII, were seen in about 33% of euthyroid relatives in a single family in the present study. This raises the possibility of a role for genetic factors in the development of ophthalmopathy.

In conclusion, it seems likely that the development of ophthalmopathy in patients with Graves’ hyperthyroidism and Hashimoto’s thyroiditis results from the concurrence of several environmental and genetic factors. Because individual families are small, the best approach to understanding the “genetic factor” would be to study large numbers of patients with thyroid autoimmunity with and without ophthalmopathy, for example looking for linkages between eye signs and selected polymorphisms (SNPs) of the CASQ1 and collagen XIII genes.

## Competing interests

All the authors declare that they have no competing interests.

## Authors’ contributions

MA participated in study design, collection of clinical data in primary writing of the manuscript. TM and HL carried out the laboratory assays and contributed to manuscript revision. BC contributed to project design and manuscript writing and revision. JW contributed to study design, collection of clinical data, manuscript writing and revisions and overall study supervision. All authors read and approved the final manuscript.
